# Intra-individual variability in cognitive performance predicts falls in older adults with chronic stroke

**DOI:** 10.1007/s40520-025-03287-y

**Published:** 2025-12-31

**Authors:** Vrinda Dimri, Jennifer C. Davis, Nárlon C. Boa Sorte Silva, Guilherme Moraes Balbim, Janice J. Eng, Teresa Liu-Ambrose

**Affiliations:** 1 Aging, Mobility, and Cognitive Health Laboratory, University of British Columbia, Vancouver, BC Canada; 2 Djavad Mowafaghian Centre for Brain Health, University of British Columbia, Vancouver, BC Canada; 3 Centre for Aging SMART at Vancouver Coastal Health, Vancouver Coastal Health Research Institute, Vancouver, BC Canada; 4 Faculty of Management, University of British Columbia Okanagan, Kelowna, BC Canada; 5 Department of Kinesiology, Health, and Applied Physiology, Concordia University, Montreal, QC Canada; 6 Department of Physical Therapy, Faculty of Medicine, University of British Columbia, Vancouver, BC Canada; 7 Djavad Mowafaghian Centre for Brain Health, c/o Liu-Ambrose Lab, 2215 Wesbrook Mall, Vancouver, BC V6T 2B5 Canada

**Keywords:** Intra-individual variability, Cognition, Reaction time, Falls, Chronic stroke

## Abstract

**Background:**

Common consequences of a stroke include impaired motor and cognitive function, with both being linked to increased falls and frailty. Intra-individual variability (IIV) of cognitive performance, which refers to the within-person trial-to-trial variation in reaction time during cognitive tasks, may be a useful predictor for falls in older adults with chronic stroke.

**Objective:**

To examine whether IIV or “traditional” reaction time (RT) measures of cognitive performance predict falls in older adults with chronic stroke.

**Methods:**

This study is a secondary analysis of a proof-of-concept randomized controlled trial (RCT) among community-dwelling adults with a history of stroke, aged 55 years and older, able to walk 6 m, and without dementia. Residualised intraindividual standard deviation (rISD) was the measure of IIV and mean RT was the “traditional” measure of performance on a computerised Stroop Task. Falls were tracked and adjudicated over six months.

**Results:**

120 participants with a mean (SD) age of 70 (8) years, and 46 (38%) female participants, experienced a mean of 0.61 (SD = 1.15) falls over 6 months. rISD for the congruent Stroop Task condition predicted falls, such that a one-unit increase was associated with 20.5% increase in fall rate.

**Conclusion:**

The findings suggest that IIV metrics may have the potential in fall risk screening post-stroke. Further research is required to evaluate whether IIV in cognitive performance can be improved via interventions such as cognitive training and physical activity.

## Introduction

Worldwide, there are over 101 million individuals who have experienced a stroke [1]. Common consequences of stroke include impaired motor function (e.g., limited mobility, poor balance, impaired gait) and impaired cognitive function (e.g., impairments in memory, executive functions, and attention). These post stroke impairments increases the risk of falls [2].

Falls are common among stroke survivors, with the incidence of falls ranging from 55% to 73% one-year post-stroke [3, 4]. Adults (>30 years) with stroke are twice as likely to fall than healthy community-dwelling adults without stroke [5]. Additionally, falls are the leading cause of mortality due to unintentional injuries in those aged >65 years [6] and are the third leading cause of chronic disability worldwide [7]. Falls and frailty have a reciprocal relationship, such that falls can lead to frailty and frailty increases the risk of falls [8]. Thus, it is important to prevent falls among older adults with stroke. Early detection of risk factors is critical in falls prevention.

To date, studies have largely identified similar fall risk factors in stroke populations as in non-stroke populations [9, 10]. However, specific risk factors, such as impaired cognitive function, may play a greater role among stroke survivors. Notably, executive functions performance was independently associated with physiological falls risk among community-dwelling older adults with chronic stroke (i.e., >12 months after stroke), after accounting for age, quadriceps strength of the paretic side, and current physical activity level [11].

Executive functions are cognitive processes that regulate one’s thoughts and behaviors while optimising goal-directed behavior and countering automatic responding [12]. Three key executive processes are: (1) set-shifting; (2) updating; and (3) response inhibition [13]. Impaired response inhibition, in particular, is associated with impaired mobility [11], balance [14, 15], and falls among stroke survivors [16].

Likewise, deficits in attention and processing speed, both of which fall under the neurocognitive domain of complex attention, are commonly observed in stroke survivors [17–20]. Evidence suggests that attentional deficits are associated with falls in older adults with chronic stroke [21]. Additionally, slower processing speed (i.e., ability to perceive, process, and respond to information) is associated with poor mobility [22] and predicts falls in older adults without stroke [23–25]. However, the relation between processing speed and falls in older adults with chronic stroke is unknown.

Previous evidence has relied on “traditional” measures of cognitive performance on neuropsychological tests such as: (1) number of correct responses; (2) time for completion; or (3) mean or median reaction time (RT). While there are recognised advantages of using these approaches, a growing body of evidence suggests that subtle alterations in cognitive processes can be detected by assessing variability of cognitive performance [26]. Research over the past decade shows intra-individual variability (IIV) in cognitive performance as a more sensitive measure of change in cognitive function than traditional measures [27]. IIV may capture aspects of cognitive functioning that would otherwise be missed by traditional measures, thereby allowing for a more complete understanding of age-and-pathology related changes in cognitive performance [26]. Unlike traditional measures, IIV assesses consistency in performance by measuring cognitive performance (e.g., reaction time) for an individual on a single task (e.g., Stroop Task), across multiple occasions (e.g., multiple trials of the task) [26, 28]. Greater variability in cognitive performance (i.e., higher IIV) in older adults is also associated with increased dementia risk [29].

Notably, higher IIV is associated with slower gait speed [30], increased fall risk [27], and prospective falls in older adults [31, 32]. A systematic review of five studies (2 prospective and 3 retrospective studies) showed a strong and significant association between high IIV and falls in older adults [33]. Whether IIV in cognitive performance, specifically in response inhibition and processing speed, is predictive of falls in older stroke survivors is unknown. Thus, we aimed to examine whether IIV or traditional RT measures of executive function and processing speed, as measured by performance on the Stroop Task, predict falls over a period of 6 months in older adults with chronic stroke.

## Methods

### Study design and setting

This study is a secondary longitudinal analysis of a 3-group parallel, single -blinded, proof-of-concept randomized controlled trial (RCT) [34]. The study protocol and the primary findings are published [34, 35]. This study included a 6-month intervention and a 6-month follow-up. The trial was conducted at a research centre located in Vancouver, British Columbia, Canada. Ethical approval was provided by the University of British Columbia Clinical Research Ethics Board (H13-00715, 26 July 2013) and Vancouver Coastal Health Research Institute (V13-00715). Informed consent was obtained from all participants. Clinical Trials Registration: NCT01916486.

### Inclusion and exclusion criteria

Eligible participants were community-dwelling adults who had an ischemic or hemorrhagic stroke. Additional inclusion criteria were: (1) aged 55 years or older; (2) had 1 or more strokes at least 12 months before study enrolment; (3) a Mini-Mental State Examination (MMSE) score of 20 out of 30 or greater at screening, including a perfect score on the 3-step command to ensure intact comprehension and ability to follow instructions [36]; (4) English-speaking; (5) not expected to start or were stable on a fixed dose of cognitive medications during the 12-month study period; (6) able to walk 6 m with rest intervals with or without assistive devices; and (7) not currently participating in any regular therapy or progressive exercise. Exclusion criteria were: (1) previous dementia diagnosis; (2) neurodegenerative disease; (3) high risk for cardiac complications; (4) usage of medications that may negatively affect cognitive performance; (5) aphasia as judged by an inability to communicate via telephone.

### Randomization and blinding

Participants were randomly allocated (2:2:3) to (1) multicomponent exercise training (EX); (2) cognitive and social enrichment activities (ENRICH); or (3) a control group consisting of balance and toning exercises (BAT). Allocation was concealed and assessors were blinded to the group allocation.

### Interventions

We use data from an RCT study that was designed to assess the effectiveness of multicomponent exercise training or cognitive and social enrichment activities on cognitive function in older adults with chronic stroke. However, the present analyses focus on assessing the predictive value of variability in cognitive performance on falls across groups. The intervention details are briefly outlined here. The multicomponent exercise training (EX) was based on the Fitness and Mobility Exercise (FAME) program and included progressive strength, aerobic, agility, and balance training exercises (fameexercise.com) [37]. The (ENRICH) program was designed based on a prior pilot study [38] and current evidence [39]. The program included computerised cognitive training and social and cognitive enrichment activities. The balance and tone (BAT) program included non-progressive stretching, deep breathing and relaxation exercises, general posture education, grip strength and dexterity exercises, and light isometric toning exercises. Additional details are provided in the published protocol [34].

### Measures

Measures were acquired at baseline, 6 months (i.e., end of intervention), and 12 months (i.e., 6-month follow-up) by blinded assessors.

### Descriptive measures

Descriptive measures included age, biological sex, number of strokes, and education. Global cognitive function was assessed using the Montreal Cognitive Assessment [40]. Fugl-Meyer Assessment Motor scale was used to assess motor function of the upper and lower extremities [41].

### Computerised Stroop task

A computerised version of the Stroop task assessed response inhibition. Color (e.g., GREEN, BLUE, RED) words and non-color words (e.g., DISK, SCREEN), presented in one of the three colors (blue, green, or yellow) were shown individually on the computer screen for a duration of 2000ms. Participants were instructed to press the response pad button that corresponded to the color of the word. Participants were instructed to respond as quickly and accurately as possible. There were 18 practice trials, followed by the task which consisted of 42 congruent trials (e.g., the word GREEN in a green font), 42 incongruent trials (e.g., the word GREEN in a yellow font), and 42 neutral trials (e.g., the word SCREEN in a green font). We derived the mean RT cost (i.e., interference score) by subtracting the congruent mean RT from the incongruent mean RT (*Mean RT incongruent - Mean RT congruent*). The mean RT cost score measures response inhibition while the congruent trials assess processing speed and attention. The cost score (i.e., *score on incongruent trial - score on congruent trial*) is considered to be a more robust measure of response inhibition than just scores on the incongruent trial, as it captures performance unbiased by differences in response time [42, 43].

### Data cleaning

We extracted the raw RT for all accurate trials and trimmed the accurate RT by removing latencies lower than 150ms or 3 standard deviations (SD) above the mean for the same participant and condition [44]. We performed imputation of the excluded data by applying a regression substitution procedure that creates individualized reaction time equations [44, 45]. These equations were then used to predict missing values. If a participant had more than 50% of data missing across trials, we did not perform imputation, and the case data were excluded. Overall, the percentage of missing data was low (0.5%; 77 out of 10,085 observations were missing).

### Computation of mean RT and IIV

For traditional measures of performance, we calculated the mean RT for each trial and condition. All mean RTs were calculated using only trials with correct responses. For IIV, we calculated the residualised intra-individual standard deviation (rISD). The rISD is an unbiased measure of variability that accounts for within (i.e., trial) and between participant (i.e., age group) sources of variation that could affect RT latencies [26, 44]. RT data, from accurate trials, for all participants was entered in a regression model with RT per trial, categorical age (i.e., 55–59, 60–64, 65–69, 70–74, 75–79, or 80–84), and an interaction term (trial × age) as model predictors (see Equation below) [44]. Next, we extracted residuals from each model and transformed these residuals into T scores. We then computed the SD of the transformed residuals (i.e., rISD) separately per participant. We computed rISD separately for the congruent and incongruent conditions in the computerised Stroop task. We derived the rISD cost (i.e., interference score) by subtracting the congruent rISD from the incongruent rISD (*rISD incongruent - rISD congruent*).

$$\begin{aligned} \:Equation\:&=a+b\left(age\:group\right)+c\left(trial\right)+d\left(age\:group\:x\:trial\right)+e \end{aligned}$$

### Falls

Our main outcome of interest was number of falls. A fall was defined as “unintentionally coming to the ground or some lower level and other than as a consequence of sustaining a violent blow, loss of consciousness, sudden onset of paralysis as in stroke or an epileptic seizure”. Falls were monitored using monthly fall diary calendars during the six-month intervention period. Participants were required to indicate the date they experienced a fall along with additional details such as injuries caused by fall, medical care received, and location of the fall (indoor vs. outdoor). Falls were adjudicated based on interviews with a blinded assessor (JCD). We also measured total exposure time, defined as the time participants were followed in the study (i.e., baseline to final assessment or withdrawal date).

### Statistical analysis

R version 4.2.0 (https://www.R-project.org/), using the packages tidyverse (version 1.3.1), lme4 (version 1.1–32.1), lmerTest (version 3.1-3.1), emmeans (version 1.7.4-1.4), knitr (version 1.39), patchwork (version 1.1.1), and mice (version 3.16.0) for predicting missing data, were used for IIV data preparation and cleaning. The primary analyses were conducted on STATA v17.0 using an available case analysis. To examine whether baseline IIV, as measured by a computerised Stroop task, predicted falls that were tracked over 6 months, we used a negative binomial regression model, an extension to the Poisson model which accommodates overdispersion and accounts for person time at risk (i.e., exposure time) for falls [46]. We ran four separate regression models with total falls at 6 months as the dependent variable. Independent variables were a) IIV, indexed as rISD, per condition (*congruent and cost*) and, b) mean RT per condition (*congruent, and cost*). Sex [47], baseline MoCA scores [48], baseline Fugl-Meyer assessment motor scale scores [49], exposure time, group allocation, and age were included as covariates. Incident rate ratio (IRR) and 95% confidence interval were calculated. Statistical significance was defined as α < 0.05. 

## Results

One hundred and twenty participants were enrolled, out of which thirty-four participants were randomised to the exercise group (EX), thirty-four to the cognitive and social enrichment group (ENRICH), and fifty-two to the balance and tone group (BAT). The mean (SD) baseline age for the total sample was 70 (8) and 46 (38%) were female participants (Table 1). The mean (SD) baseline MOCA score was 21.89 (4.15), indicating that the participants had mild cognitive impairment [40]. The mean (SD) baseline Fugl-Meyer Assessment Motor score was 81.06 (22.98), indicating moderate motor impairment [50].

**Table 1:** Sample characteristics at baseline

Variables	Total sample, mean (SD), *n* = 120	Range
Age, years	70 (8)	55 to 90
*Biological sex, n (%)*		
Male	74 (62%)	-
Female	46 (38%)	-
Height, cm	167.37 (9.80)	148.20 to 186.37
Weight, kg	77.95 (15.85)	40.7 to 122.40
Body mass index (kg/m2)^a^	27.68 (4.43)	16.35 to 47.74
*Education, n (%)*		
High school diploma or less	24 (20%)	-
Some college or university	36 (30%)	-
University degree or higher	60 (50%)	-
Number of strokes	1.19 (0.57)	1 to 4
Number of falls in past year	1.24 (2.90)	0 to 25
Functional comorbidity index (0–18)^b^	3.41 (1.79)	1 to 9
Montreal cognitive assessment (0–30)^c^	21.89 (4.15)	10 to 30
Fugl-Meyer assessment motor score (0–100)^d^	81.06 (22.98)	6 to 100
Short physical performance battery (0–12)	8.18 (2.67)	1 to 12
*Stroop task, mean RT, ms*		
Congruent condition	1034.56 (181.35)	647.51 to 1546.47
Incongruent condition	1184.96 (194.52)	721.96 to 1648.50
Cost score	159.33 (99.71)	−5.93 to 468.66
*Stroop task, rISD*		
Congruent condition	7.65 (2.31)	3.57 to 12.80
Incongruent condition	8.04 (1.98)	4.19 to 13.35
Cost score	0.45 (2.25)	−5.22 to 6.13

^a^Body mass index is calculated as weight in kilograms divided by height in meters squared

^b^The Functional Comorbidity Index ranges from 0 (no comorbid illness) to 18 (to highest number of comorbid illness). Higher scores are associated with lower physical function

^c^The Montreal Cognitive Assessment ranges from 0 (lowest) to 30 (highest); scores 26 to 30 are the reference range

^d^The Fugl-Meyer Assessment Motor Score ranges from 0 (very severe impairment) to 100 (no impairment); scores greater than 79 are indicative of mild motor impairment, 56 to 79 indicate moderate impairment, 36 to 55 indicate severe impairment, and 0 to 3 indicate very severe impairment

^e^The Short Physical Performance Battery ranges from 0 to 12; scores lower than 9 indicate increased risk of disability

Falls counts and exposure time are detailed in Table 2. One participant withdrew from the study immediately following baseline assessment, due to which no falls data were collected. Hence, the prospective falls data is reported for 119 participants. Over a mean (SD) follow-up of 192 (± 49) days, 34% of participants reported 1 or more falls.

**Table 2:** Prospective falls counts

Falls Count	Mean (SD) or *n* (%)	Range
Total exposure time (days)	192.32 (48.78)	21 to 246*
Exposure time to first fall (days)	46.56 (24.13)	1 to 89
Number of falls	0.61 (1.15)	0 to 6
0	79 (66.4%)	-
1	24 (20.2%)	-
2	8 (6.7%)	-
3	3 (2.5%)	-
4	2 (1.7%)	-
5	2 (1.7%)	-
6	1 (0.8%)	-

*For 11 (9.2%) participants, exposure time was shorter than intervention duration due to attrition after baseline assessment. Final assessment was delayed for 75 (62.5%) participants due to personal circumstances

### Mean RT and falls

The results of the negative binomial regression models which assess whether baseline mean reaction time predicts falls prospectively are reported in Table 3. Mean RT cost scores (IRR = 1.003; 95% CI [0.999–1.006]; *p* = 0.143), and mean RT for congruent condition (IRR = 1.000; 95% CI [0.998–1.003]; *p* = 0.743) did not predict falls, while holding all other variables in the model constant.

### IIV and falls

Residualised ISD for the congruent condition, significantly predicted falls prospectively (IRR = 1.205; 95% CI [1.023–1.420]; *p* = 0.025). This is the estimated rate ratio for a 1-unit increase in the IIV score, given the other variables are held constant in the model (Table 4). If an individual’s rISD score increases by a 1-unit equivalent, their rate for total falls would increase by a factor of 20.5%, while holding other variables in the model constant. The rISD cost scores did not predict falls (IRR = 0.975; 95% CI [0.836–1.137]; *p* = 0.748).

**Table 3:** Negative binomial regression model for mean reaction time and incidence rate ratio for falls

Variable	Incidence rate ratio	Standard error	*P*>|z|	95% Confidence Interval
***Congruent Condition (n = 118)***				
Mean RT	1.000	0.001	0.743	0.998–1.003
Total exposure time	1.004	0.004	0.357	0.996–1.012
Group				
*ENRICH*	0.909	0.386	0.823	0.396–2.090
*EX*	1.004	0.429	0.993	0.434–2.319
MoCA	0.999	0.053	0.980	0.900–1.108.900.108
Fugl-Meyer	1.016	0.010	0.092	0.997–1.035
Biological sex	1.009	0.379	0.981	0.483–2.106
Age	0.982	0.022	0.428	0.940–1.026
***Cost (Incongruent – Congruent) (n = 115)***				
Mean RT	1.003	0.002	0.143	0.999–1.006
Total exposure time	1.003	0.004	0.628	0.994–1.011
*Group*				
*ENRICH*	0.918	0.378	0.835	0.409–2.057
*EX*	0.794	0.353	0.604	0.336–1.900.336.900
MoCA	1.014	0.055	0.791	0.913–1.127
Fugl-Meyer	1.011	0.009	0.222	0.993–1.030
Biological sex	0.983	0.367	0.964	0.473–2.045
Age	0.974	0.022	0.242	0.932–1.018

RT (reaction time); ENRICH (cognitive and social enrichment group); EX (multicomponent exercise group); MoCA (Montreal Cognitive Assessment)

**Table 4:** Negative binomial regression model for residualised intra-individual standard deviation and incidence rate ratio for falls

Variable	Incidence rate ratio	Standard error	*P*>|z|	95% Confidence Interval
***Congruent Condition (n = 119)***				
Residualised ISD	1.205	0.101	0.025*	1.023–1.420
Total exposure time	1.007	0.005	0.164	0.997–1.016
Group				
*ENRICH*	0.781	0.334	0.564	0.338–1.805
*EX*	1.105	0.465	0.812	0.485–2.519
MoCA	1.023	0.052	0.657	0.925–1.130
Fugl-Meyer	1.019	0.009	0.048	1.000–1.038.000.038
Biological sex	1.236	0.467	0.575	0.589–2.594
Age	0.972	0.022	0.207	0.930–1.016
***Cost (Incongruent – Congruent) (n = 115)***				
Residualised ISD	0.975	0.077	0.748	0.836–1.137
Total exposure time	1.004	0.004	0.399	0.995–1.012
*Group*				
*ENRICH*	0.906	0.379	0.814	0.398–2.060
*EX*	0.798	0.359	0.616	0.331–1.927
MoCA	1.026	0.056	0.635	0.923–1.140
Fugl-Meyer	1.012	0.009	0.201	0.994–1.030
Biological sex	1.122	0.419	0.757	0.540–2.332
Age	0.983	0.021	0.422	0.941–1.026

*Significance level set at alpha < 0.05. Abbreviations: RT (reaction time); ENRICH (cognitive and social enrichment group); EX (multicomponent exercise group); MoCA (Montreal Cognitive Assessment)

## Discussion

The study examined whether IIV or traditional RT measures of cognitive performance predict falls in older adults with chronic stroke. Our findings indicated that IIV in processing speed, indexed as higher IIV on congruent trials of the Stroop Task, significantly predicted falls over 6 months in older adults with chronic stroke, while holding other variables constant. Specifically, a higher IIV score on the congruent condition of the Stroop task, measuring attention and processing speed, was associated with a 20.5% increase in fall rate. Our findings concur with previous studies involving older adults, which have shown a significant association between higher IIV in cognitive performance and increased falls [31–33]. In particular, previous evidence shows that frequent fallers perform poorly than healthy controls on attention tasks, as measured by variability in RT during the congruent condition of the Stroop task [51].

To our knowledge, this is the first study to evaluate whether IIV in processing speed and attention predicts falls in older adults with chronic stroke. Impairment in executive functions, processing speed, and attention are commonly observed in post-stroke [20, 52]. Moreover, impaired cognitive function serves as a key risk factor for falls [2, 11, 14, 16].

Interestingly, in our study, the traditional measure of cognitive performance (i.e., mean RT) did not predict falls in older adults with chronic stroke. These findings highlight that IIV might be a more sensitive measure of change in processing speed as compared with traditional measures [27]. This is in line with previous evidence, suggesting IIV to be a unique and useful measure of cognitive performance [26, 28, 44]. Overall, our findings indicate that by capturing fluctuations in processing speed, IIV metrics may have the potential in augmenting current falls risk screening post stroke.

Contrary to previous evidence [16], mean RT in response inhibition did not predict falls in older adults with chronic stroke. We speculate that the lack of association could be due differences in the participants efforts and motivation while performing more complex cognitive tasks (response inhibition) than simple tasks (attention and processing speed). It is likely that the participants paid more attention to the complex incongruent trials in order to improve task performance. This can also be observed in the mean RT scores, where we found no significant difference between incongruent and congruent trials. Additionally, in a study with previous fallers, processing speed was found to be predictive of falls whereas response inhibition did not predict falls [23]. In our study, approximately 44% (*n* = 52) of older adults with chronic stroke had experienced at least one fall in the past year.

The study has certain limitations. First, the study sample included chronic stroke survivors with mild-to-moderate motor impairment, which could have played a role in increasing falls risk. While we did account for motor impairments by including the Fugl-Meyer Motor Assessment scores, we cannot fully attribute the increase in falls to higher variability in cognitive performance. Evidence suggests that impaired processing speed and attentional deficits, including increased mental distraction and difficulty multi-tasking, can negatively impact performance on motor tasks (e.g., timed-up and go), postural balance and gait [21, 22].Thus, factors such as gait speed and balance need to be considered while ascertaining falls risk. Our results are also limited to older adults with stroke with mild-to-moderate cognitive and motor impairment. Therefore, these results cannot be generalized to those with more severe stroke-related impairments. Our sample primarily consisted of highly educated individuals with the majority being male, thereby limiting the generalisability of the results. To minimise the effect of sex, we included it in our models as a covariate. Finally, we conducted a secondary analysis using data from an RCT, which was powered to assess the intervention effects, which increases the probability of observing a Type 1 error. Thus, the results must be interpreted with caution.

In conclusion, higher IIV in processing speed predicted falls in older adults with chronic stroke. Additionally, traditional measures of cognitive performance did not predict falls. This suggests that IIV or variability in cognitive performance might be a useful metric to screen for fall risks in stroke populations. Further research is required to evaluate whether IIV in other cognitive domains are associated with falls and whether IIV in cognitive performance can be improved (i.e., reduced) in older adults with chronic stroke via certain interventions, such as cognitive training or physical activity [43]. Finally, given the underlying neurological basis of a stroke, it is crucial to explore the potential neurophysiological correlates of IIV and falls. Prior research has linked higher IIV to greater white matter hyperintensity burden [53], reduced white matter integrity [54], and potential neural network dysfunction [55]; all of which have been shown to be associated with increased falls [56–58]. Thus, further research examining the potential relationship between IIV, falls, and brain structure and function is warranted.

## Data Availability

The data supporting the findings of this study are available on request from the corresponding author. The data are not publicly available due to privacy or ethical restrictions.
